# The ClinicalTrials.gov Landscape of Multiple Myeloma Clinical Trials: A 20-Year Analysis of Geographic Distribution and Growth Patterns: USMIRC Analysis

**DOI:** 10.3390/curroncol33070396

**Published:** 2026-07-01

**Authors:** Anas Zayad, Osama Younis, Carmel Awadallah, Ishita Kamboj, Abdelrhman Mohammed, Ahmad E. Shatnawi, Amr Ali, Hamed Alzatary, Abdullah Mohammad Khan, Hira Shaikh, Omar Alkharabsheh, Mansi R. Shah, Prerna Mewawalla, Joseph P. McGuirk, Zahra Mahmoudjafari, Muhammad Umair Mushtaq, Jeries Kort, Alma Habib, Shebli Atrash, Al-Ola Abdallah

**Affiliations:** 1 Department of Internal Medicine, Hamad Medical Corporation, Doha 3050, Qatar; 2 School of Medicine, University of Jordan, Amman 11942, Jordan; 3 Division of Internal Medicine, St John Episcopal Hospital, Far Rockaway, NY 11691, USA; cawadallah@ehs.org; 4 Department of Internal Medicine, Prime South GME—St. Joseph Medical Center, Kansas City, MO 64114, USA; ikamboj@primehealthcare.com; 5 Division of Hematology/Oncology, University of Alabama at Birmingham, Birmingham, AL 35294, USA; 6 Faculty of Medicine, Jordan University of Science and Technology, Irbid 22110, Jordan; aeshatnawi21@med.just.edu.jo; 7 Faculty of Medicine, American University of Beirut, Beirut 1107 2020, Lebanon; ama240@mail.aub.edu; 8 Internal Medicine, Wellington Regional Medical Center, Wellington, FL 33414, USA; hamed.alzatary@uhsinc.com; 9 Division of Hematology, The Ohio State University, Columbus, OH 43210, USA; 10 Division of Hematology, Oncology, and Blood & Marrow Transplantation, University of Iowa, Iowa City, IA 52242, USA; 11 Division of Hematology and Oncology, University of Cincinnati, Cincinnati, OH 45221, USA; alkharor@ucmail.uc.edu; 12 Division of Hematology, Rutgers Cancer Institute of New Jersey, New Brunswick, NJ 08901, USA; 13 Division of Hematology and Cellular Therapy, Allegheny Health Network Cancer Institute, Pittsburgh, PA 15224, USA; 14 Division of Hematologic Malignancies & Cellular Therapeutics, University of Kansas Medical Center, Westwood, KS 66160, USA; jmcguirk@kumc.edu (J.P.M.);; 15 Levine Cancer Institute, Atrium Health Wake Forest University School of Medicine, Charlotte, NC 28204, USA

**Keywords:** multiple myeloma, clinical trials, geographic distribution, global health, health disparities, OECD, world bank income classification, population-adjusted trial density, international collaboration, oncology research equity

## Abstract

Background: Multiple myeloma (MM) has experienced rapid therapeutic innovation over the past two decades, leading to a substantial increase in clinical trial activity. However, the geographic distribution of these trials and the representation of different economic regions remain poorly characterized. We evaluated the global distribution, growth patterns, and phase-specific trends of MM clinical trials and trial sites across different economic settings. Methods: We conducted a retrospective registry-based analysis interventional MM clinical trials registered on ClinicalTrials.gov between January 2006 and January 2026. Trials were categorized based on the economic classification of participating countries using World Bank income groups and Economic Co-operation and Development (OECD) status. Trial characteristics including phase, geographic distribution, number of participating sites, and site-years were analyzed. Population-adjusted trial density and compound annual growth rates (CAGR) were calculated to assess temporal trends and geographic representation. Results: A total of 845 interventional MM clinical trials were identified during the study period. Trial activity was highest in the United States (337 trials, 39.9%), followed by international trials (271, 32.1%), high-income-OECD countries (129, 15.3%), and upper-middle-income countries (103, 12.2%), while high-income non-OECD countries contributed only a small fraction of trials. Trial activity increased substantially over time across all regions with the highest growth observed in upper-middle-income countries (CAGR 18.5%). The US demonstrated the highest population-adjusted trial density (0.99 per million population) and accounted for the largest number of trial sites and site-years. Phase-specific analyses revealed distinct geographic patterns. Phase 1 trials were predominantly conducted in the US and in international collaborative trials. Phase 3 trials were largely international, although the majority of participating sites remained located in the US and High-income countries that are members of the OECD (HIC-OECD). Conclusions: Over the past two decades, MM clinical trial activity has expanded globally but remains highly concentrated in the United States and high income-OECD countries, particularly with respect to trial sites and population-adjusted trial density. Although upper-middle-income countries have shown the fastest growth in trial activity expanding clinical trial infrastructure and strengthening international collaboration will be essential to promote a more equitable global distribution of MM research.

## Simple Summary

Multiple myeloma is a blood cancer that has undergone major treatment advances over the past two decades, leading to a rapid increase in clinical research worldwide. However, it remains unclear whether opportunities to participate in clinical trials are distributed equally across different countries and regions. In this study, we examined the ClinicalTrials.gov patterns of multiple myeloma clinical trials registered over a 20-year period, focusing on where trials were conducted, how research activity changed over time, and how participation differed according to national economic status. We found that most clinical trials and research sites were concentrated in the United States and other high-income countries, while lower-income regions remained underrepresented despite recent growth in research activity. International collaboration increased substantially over time, but research infrastructure remained unevenly distributed. These findings highlight important global disparities in access to clinical research and emerging therapies. Expanding clinical trial infrastructure and strengthening international collaboration may help ensure that future advances in multiple myeloma benefit patients more equitably around the world.

## 1. Introduction

Multiple myeloma (MM) has undergone remarkable therapeutic transformation over the past two decades, evolving from a disease with limited treatment options to one characterized by rapidly expanding therapeutic innovation including proteasome inhibitors, immunomodulatory agents, monoclonal antibodies, bispecific T-cell engagers, and cellular therapies [1,2]. As a result, MM has become one of the most intensively investigated hematologic malignancies, with a substantial increase in global clinical trial activity.

Despite these advances, MM continues to impose a growing global health burden. Global Cancer Incidence, Mortality and Prevalence database (GLOBOCAN) 2020 estimates reported approximately 176,000 new cases worldwide, with substantial geographic variation in both incidence and mortality [3]. Importantly, mortality rates remain disproportionately higher in low- and middle-income countries (LMICs), likely reflecting differences in diagnostic capacity, access to modern therapies, and health system infrastructure. These disparities highlight an increasingly recognized challenge in oncology: the rapid pace of therapeutic innovation has not been matched by equitable global access to emerging treatments.

Although MM is not the most prevalent malignancy, it has become one of the most rapidly evolving fields in hematologic oncology, driven by successive generations of targeted agents and immunotherapies [4]. Parallel to the therapeutic evolution of MM, clinical trial activity has expanded considerably. A multi-registry analysis integrating ClinicalTrials.gov, the European Union Clinical Trials Register, and the Japan Primary Registries Network identified 1879 MM clinical trials, exceeding the number of trials conducted in several other malignancies, including ovarian cancer and acute myeloid leukemia [5]. Given the comparatively lower global incidence of MM relative to many solid tumors, this finding suggests a high concentration of research activity relative to disease prevalence, reflecting strong engagement from both academic investigators and the pharmaceutical industry. However, the geographic distribution of this research activity remains poorly characterized [6].

Emerging evidence suggests that the globalization of patient enrollment in oncology trials has not been accompanied by comparable globalization of trial leadership or research infrastructure. Analyses of trials supporting United States Food and Drug Administration approvals indicate that pivotal oncology trials are predominantly led by investigators and institutions in high-income countries (HICs), with limited participation from upper-middle-income countries and minimal representation from lower-income settings [7]. In MM, this imbalance is particularly consequential because participation in clinical trials often represents the earliest pathway for patient access to novel therapeutics and may influence subsequent regulatory approvals, drug availability, and clinical adoption.

Understanding how clinical research activity is distributed globally is therefore essential not only for ensuring equitable participation in therapeutic innovation, but also for improving the generalizability of trial results across diverse patient populations and health systems. To our knowledge, this is the first study to comprehensively characterize the global multiple myeloma clinical trial landscape, integrating geographic distribution, trial-site density, economic classification, funding patterns, and phase-specific trends over a two-decade period using ClinicalTrials.gov data.

In this study, we sought to characterize the global landscape of MM clinical research over the past two decades. Specifically, we evaluated the geographic distribution and temporal trends of MM clinical trials and participating trial sites, examining patterns across countries, economic classifications, and trial phases. We further quantified population-adjusted trial and site density to better understand how MM clinical research infrastructure is distributed worldwide and how these patterns have evolved over time.

## 2. Methods

### 2.1. Study Design and Data Source

This retrospective, registry-based descriptive study used data from the ClinicalTrials.gov database. A comprehensive search was performed to identify MM interventional trials registered between 1 January 2000, and 31 January 2026.

### 2.2. Trial Characteristics and Data Extraction

Trials were identified using condition-based search terms related to “multiple myeloma.” Eligible studies included phase 1–3 interventional trials in which MM was the primary disease and recruitment status was completed, recruiting, or active (including active but not recruiting) at the time of data extraction. Trials were excluded if they were incomplete, terminated, withdrawn, follow-up studies, secondary analyses, or not primarily focus on MM.

To ensure consistency in registry reporting, analyses were restricted to trials registered between 2006 and 2026 because trial registration was limited before 2006 and increased substantially following broader adoption of registry reporting [8]. For each eligible study, the following variables were extracted: year of registration, trial phase, recruitment status, sponsor type, enrollment size, number of trial sites, and country or countries of trial conduct.

### 2.3. Geographic Classification

Trials were categorized as single-country or international (≥2 countries being involved in the trial). Trial sites represented the number of participating study locations reported for each trial. To capture trial scale and duration, trial site–years were calculated as the number of trial sites within a country multiplied by the duration of trial activity in years.

Countries were classified using the World Bank income classification system based on gross national income per capita calculated using the Atlas method [9]. Countries were categorized as low-income, lower-middle-income, upper-middle-income, or high-income. High-income countries were further stratified by Organisation for Economic Co-operation and Development (OECD) membership at the time of analysis (HIC-OECD vs. HIC–non-OECD) [10]. Given the substantially higher number of MM trials conducted in the United States, the US was analyzed separately. For international trials, income-based stratification was not applied. Income classification thresholds are provided in Supplementary Table S1. Countries were categorized according to the World Bank Country and Lending Groups classification, and the corresponding list of countries within each income category is available from the World Bank [9].

### 2.4. Outcomes and Statistical Analysis

Primary outcomes included number of trials, trial density per million population, and compound annual growth rate (CAGR) of trial activity. Secondary outcomes assessed clinical trial infrastructure, including number of trial sites and trial site–years. Population-adjusted metrics were calculated using 2025 United Nations population estimates [11,12].

Temporal growth rates were estimated using an exponential growth model, with logarithmic transformation and linear regression used to derive annual growth rates and 95% confidence intervals. Analyses were performed using Python version 3.13 (pandas, NumPy, and Matplotlib). Because this study used publicly available de-identified registry data, institutional review board approval was not required.

## 3. Results

### 3.1. Clinical Trial Distribution by Economic Status

**Figure 1:**
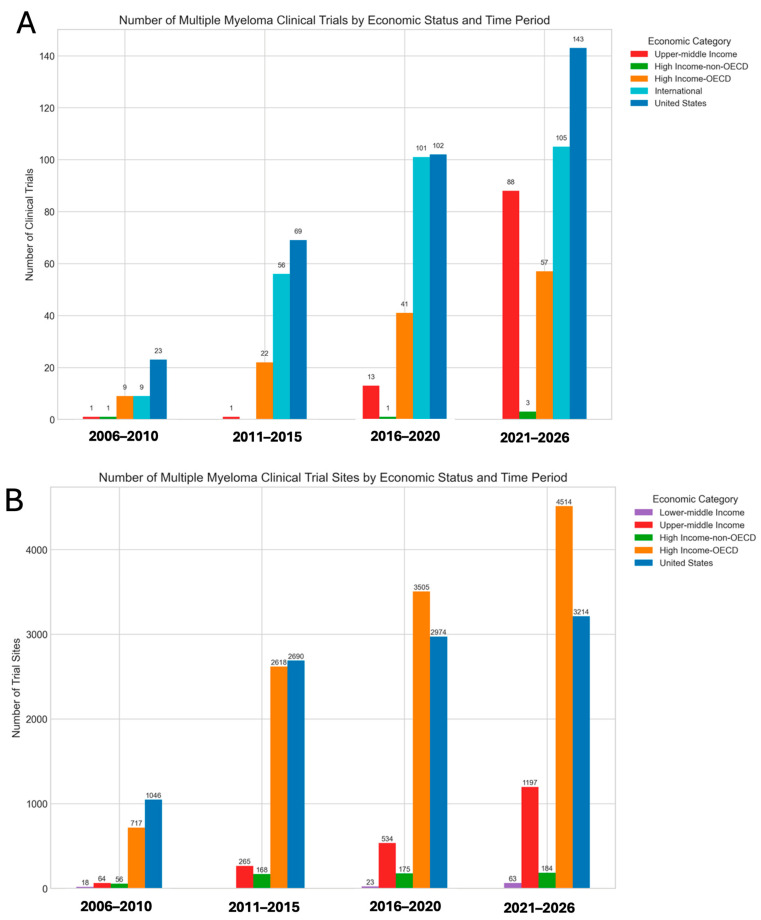
Distribution of multiple myeloma (MM) clinical trials (**A**) and clinical trial sites (**B**) by economic category across four time periods. Economic categories include low-income countries, upper-middle-income countries, high-income non-OECD countries, high-income OECD countries, international trials ((**A**) only), and the United States (US). Bars represent the number of trials initiated (**A**) and participating trial sites (**B**) in each period.

A total of 845 MM clinical trials were included (Table 1; Figure 1A,B). International trials were analyzed as a separate category based on study design. Trial activity was most concentrated in the US, which contributed 337 trials (39.9%), with a trial density of 0.99 per million population and a CAGR of 9.7%. International trials accounted for 271 trials (32.1%) with a CAGR of 9.8%. Among income-stratified categories, HIC-OECD countries contributed 129 trials (15.3%) (trial density 0.16 per million population; CAGR = 8.5%). Upper-middle-income countries (UMICs) accounted for 103 trials (12.2%), with a lower trial density (0.04 per million population) but the highest relative growth (CAGR = 18.5%). In contrast, trial representation in HIC-non-OECD countries (5 trials; 0.6%) and Low- and middle-income countries (LMICs) contributed no trials. Figure 1A shows a substantial increase in global MM clinical trial activity over time. The US consistently accounted for the largest number of trials across all periods, while international trials also increased substantially, particularly after 2016. Trials conducted in UMICs increased from 1 trial in 2006–2010 to 88 trials in 2021–2026. Other economic categories also demonstrated gradual increases in trial activity over time, although their contributions remained smaller.

Table 2 summarizes the distribution of trial sites and study years by economic classification. Overall, more than 24,000 clinical trial sites were identified globally, of which 9924 (41.31%) were located in the US, representing 41.31% of all sites, but contributed a disproportionately larger share of trial site years (105,262; 53.398%), reflecting a longer trial activity duration and higher site intensity. The US also demonstrated the highest average annual site density (14.664 sites per million population), substantially exceeding that of all the other groups. Among income-stratified categories, HIC-OECD countries accounted for the largest proportion of non-US trial sites (11,354 sites; 47.26%) and 74,165 site years (37.62%), with an average annual density of 4.47 sites per million. In contrast, UMICs contributed a modest proportion of sites and site–years, with substantially lower site density. HIC-non-OECD countries and LMICs had limited representation, with very low numbers of sites and years. Figure 1B shows a steady increase in the number of MM clinical trial sites over time across all economic categories. The US had the largest number of sites before 2016, but after 2016, the highest number of clinical trial sites was observed in HIC-OECD countries (rising from 717 sites in the earliest period to 4514 sites). Although the US remained the leading country in terms of the number of trials, it was not consistently the leading contributor in terms of trial sites.

### 3.2. Funding of Multiple Myeloma Clinical Trials and Trial Sites

**Figure 2:**
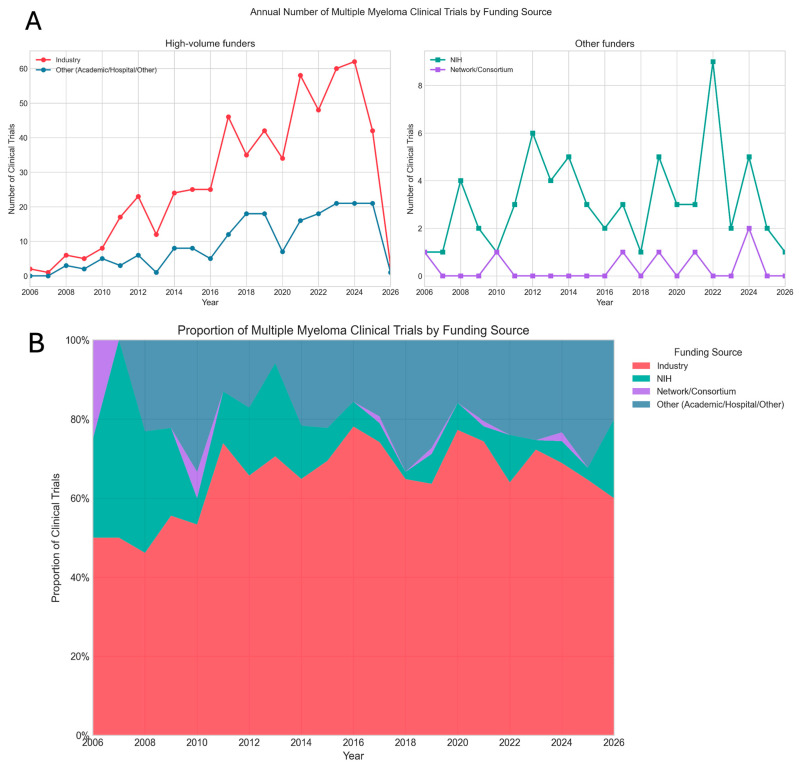
Annual trends in funding sources of multiple myeloma (MM) clinical trials from 2006 to 2026. Panel (**A**) shows the annual number of trials by funding source, and panel (**B**) shows the proportional contribution of each funding category per year. Funding categories include industry sponsors, the National Institutes of Health (NIH), cooperative networks or consortia, and other non-industry sources (academic, hospital-based, or investigator-initiated).

At the beginning of the study period, MM clinical trials were supported by a heterogeneous mix of sponsors. Over time, however, a clear shift toward industry dominance was observed. As shown in Figure 2A,B, industry-funded trials increased steadily from the mid-2000s, with marked acceleration after 2015 and peak activity in the early 2020s. A temporary decline in trial registrations was observed during 2020–2021 during the COVID-19 period, particularly among industry-sponsored studies. Non-industry trials supported by academic or hospital-based investigators also increased modestly but remained consistently fewer than industry-funded trials. In contrast, NIH-funded studies and cooperative network or consortium trials were infrequent throughout the study period, contributing only a small proportion of the total trials with notable year-to-year variability.

**Figure 3:**
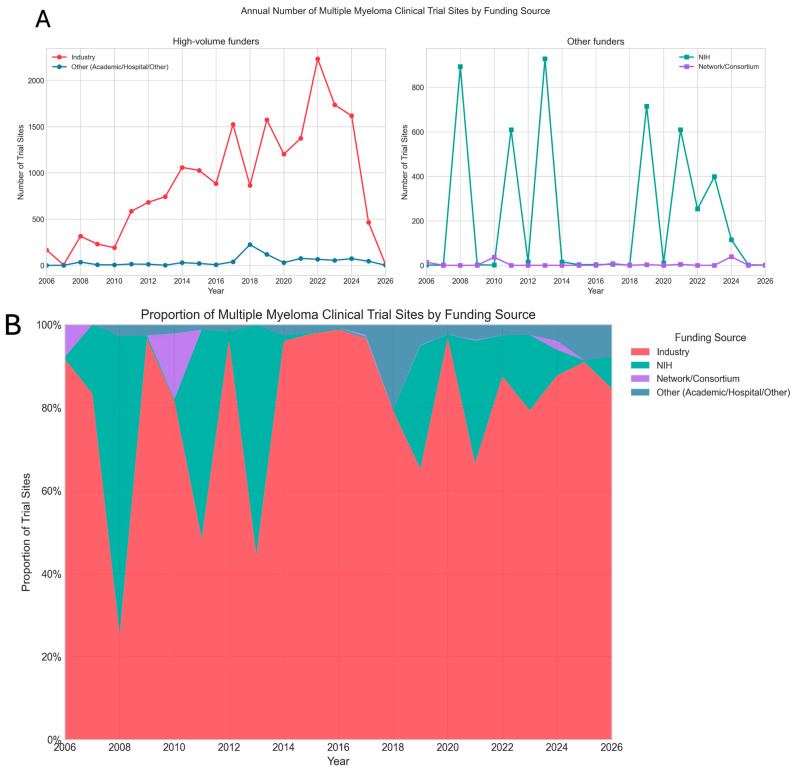
Annual trends in clinical trial sites of multiple myeloma (MM) trials by funding source from 2006 to 2026. Panel (**A**) shows the annual number of participating trial sites, and panel (**B**) shows the proportional contribution of each funding category per year. Funding sources include industry sponsors, the National Institutes of Health (NIH), cooperative networks or consortia, and other non-industry sponsors (academic, hospital-based, or investigator-initiated).

A similar pattern was observed when examining clinical trial sites. As shown in Figure 3A,B, the number of industry-supported trial sites expanded substantially over time, particularly after the early 2010s, and accounted for the majority of MM trial sites across most years, frequently exceeding 80% of all participating sites. Although site numbers peaked in the early 2020s, a decline was again observed in the most recent years. In contrast, trial sites supported by academic or other non-industry sponsors remained relatively limited, whereas NIH-funded and cooperative network-supported sites showed only intermittent activity without sustained growth.

### 3.3. Trial Density

#### 3.3.1. Trial Density by Country

**Figure 4:**
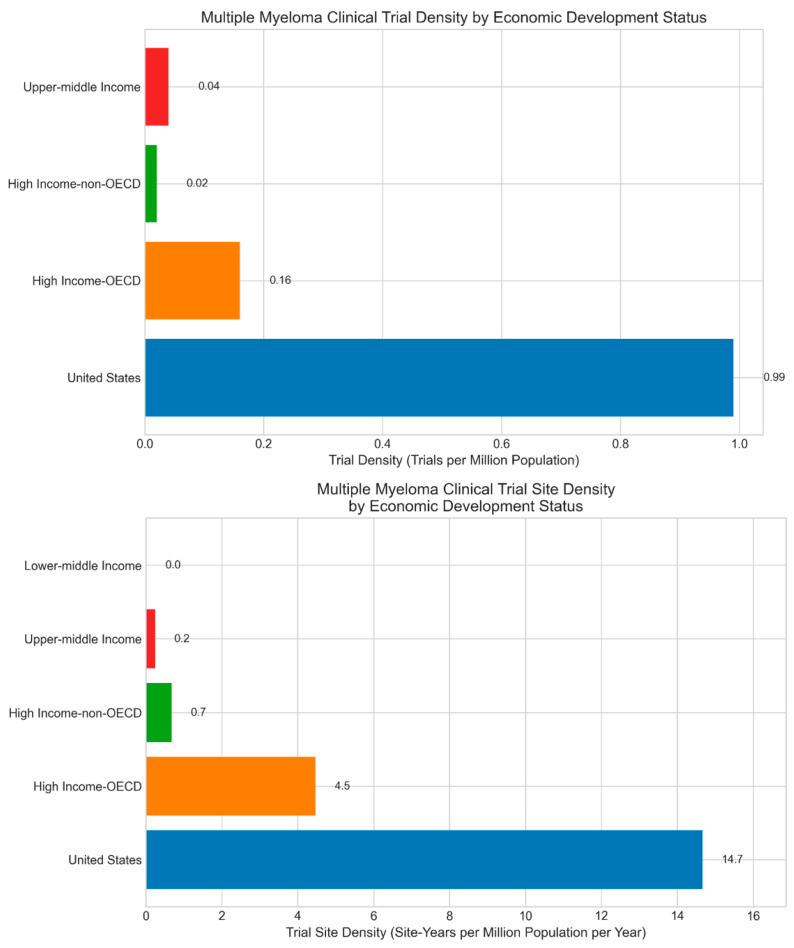
Bar plot showing population-adjusted multiple myeloma clinical trial site density, expressed as trial site–years per million population per year, stratified by World Bank economic classification, with the United States analyzed separately. Site density was calculated using cumulative trial site years over the study period.

Table 3 summarizes trial density by country, defined as the number of MM clinical trials per million population. Substantial heterogeneity in trial density was observed across countries, even within the same economic classification. The US demonstrated the highest trial density (0.99 trials per million) and accounted for the largest number of trials and patient enrollment. Several HIC-OECD countries showed relatively high trial densities despite smaller absolute trial numbers, including Israel (0.60), Norway (0.54), Denmark (0.50), and Greece (0.49). Other HIC-OECD countries, such as Switzerland, Canada, the Netherlands, and France, also demonstrated moderate trial densities. In contrast, China, despite contributing a large number of trials in absolute terms (102 trials), had a relatively low trial density (0.07) due to its large population size. A summary of trial density by economic category is shown in Figure 4.

#### 3.3.2. Trial Density by Sites

Table 4 presents the distribution of clinical trial site density by country, expressed as the average annual number of sites per million population. The US had the highest site density (14.66 sites per million), indicating the largest concentration of participating MM trials relative to population size. Several HIC-OECD countries also demonstrated substantial site density, including Israel (12.66), Denmark (9.21), Belgium (9.15), and Greece (8.85). Other HIC-OECD countries, such as the Czech Republic, Spain, Australia, Norway, and Sweden, also showed notable site densities, although at lower levels, the highest site density was predominantly observed in high-income countries, while representation from other economic groups was limited. A summary of trial site densities by economic category is shown in Figure 4.

### 3.4. Phases Distribution of Multiple Myeloma Clinical Trials and Trial Sites

#### 3.4.1. Phase 1 Clinical Trials

**Figure 5:**
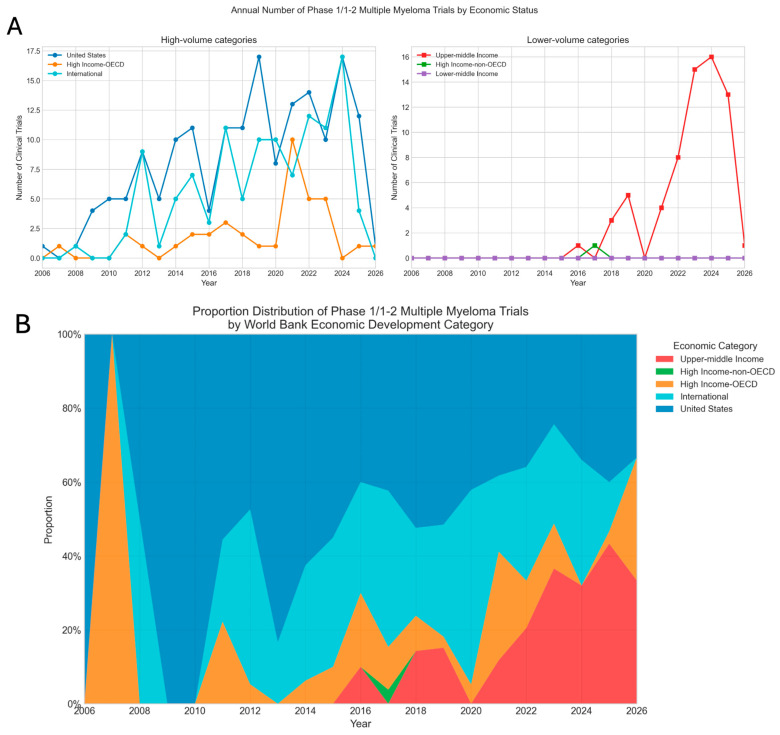
Annual trends in phase 1 multiple myeloma (MM) clinical trials from 2006 to 2026 by economic category. Panel (**A**) shows the annual number of trials, and panel (**B**) shows the proportional contribution of each category per year. Economic categories include the United States (US), high-income OECD countries (HIC-OECD), high-income non-OECD countries (HIC-non-OECD), international trials, and upper-middle-income countries (UMIC).

Figure 5A shows the annual number of phase 1 MM clinical trials by economic category, grouped into high-volume and lower-volume groups. Early-phase trials were initially dominated by the US, which consistently reported the highest number of trials during the early years of the study period. Over time, international trials increased steadily and eventually reached levels comparable to those in the US in the later years. A temporary decline in trial activity was observed around the COVID-19 period, after which trial numbers recovered. HIC-OECD countries contributed fewer early-phase trials during the earlier years but showed a noticeable increase in the later period. Among the lower-volume categories, UMICs demonstrated limited activity initially but showed a clear increase after 2016, with several trials reported in the early 2020s. In contrast, HIC-non-OECD contributed minimally throughout the study period.

Figure 5B shows the proportional distribution of phase 1 MM clinical trials across economic categories over time. The US accounted for the largest proportion of early-phase trials during the early study years, reflecting its leading role in early drug development. Over time, international trials increased steadily, reaching levels comparable to or exceeding those in the US in several later years. The contribution of HIC-OECD countries increased in the later years, indicating the broader participation of high-income countries in early-phase trials. In contrast, UMICs began contributing only after the mid-2010s, with a gradual increase toward the end of the study period. HIC-non-OECD accounted for only a very small proportion of early-phase trials throughout the study period.

#### 3.4.2. Phase 2 Clinical Trials and Trial Sites

**Figure 6:**
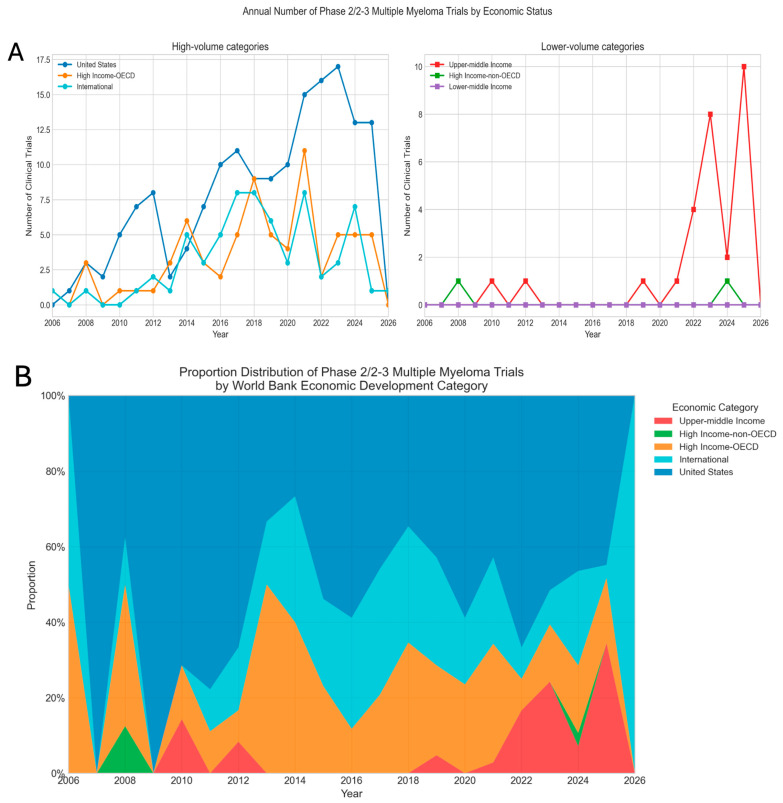
Annual trends in phase 2 multiple myeloma (MM) clinical trials from 2006 to 2026 by economic category. Panel (**A**) shows the annual number of trials, and panel (**B**) shows the proportional contribution of each category per year. Economic categories include the United States (US), high-income OECD countries (HIC-OECD), high-income non-OECD countries (HIC-non-OECD), international trials, and upper-middle-income countries (UMIC).

**Figure 7:**
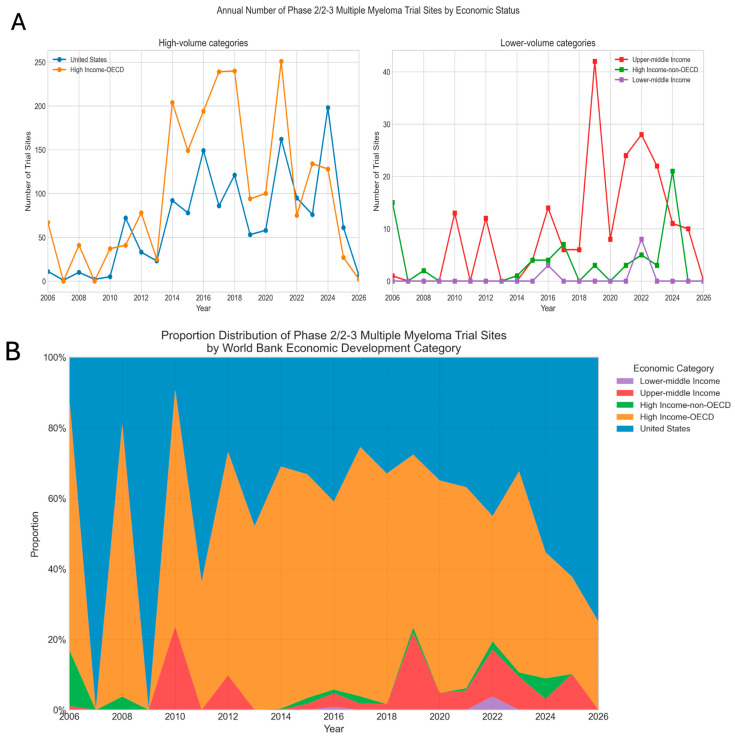
Annual trends in clinical trial sites supporting phase 2 multiple myeloma (MM) trials from 2006 to 2026 by economic category. Panel (**A**) shows the annual number of participating trial sites, and panel (**B**) shows the proportional contribution of each category per year. Economic categories include the United States (US), high-income OECD countries (HIC-OECD), high-income non-OECD countries (HIC-non-OECD), upper-middle-income countries (UMIC), and lower-middle-income countries (LMIC).

Figure 6 and Figure 7 show the trends in phase 2 MM clinical trials and trial sites across economic categories over time. As shown in Figure 6A,B, the US consistently contributed the largest number of phase 2 trials, with a gradual increase beginning in the early 2010s and peaking in the early 2020s. International trials also increased over time, particularly after the mid-2010s. Compared with the pattern observed in phase 1 trials, HIC-OECD countries had a greater contribution in phase 2 trials, with a steady rise in later years reaching levels comparable to international trials in several periods. Among the lower-volume categories, UMICs showed minimal activity during the early years but increased noticeably after 2020, with several trials reported in the most recent years.

A similar pattern was observed when examining clinical trial sites (Figure 7A,B). The number of trial sites increased substantially over time, with HIC-OECD countries contributing the largest number of sites in several years, particularly after 2014. The US also showed substantial growth in participating sites, reflecting the expansion of multi-center phase 2 trials. In contrast, UMICs demonstrated intermittent increases in trial sites during the later years, while HIC-non-OECD contributed only a small number of sites throughout the study period.

#### 3.4.3. Phase 3 Clinical Trials and Trial Sites

**Figure 8:**
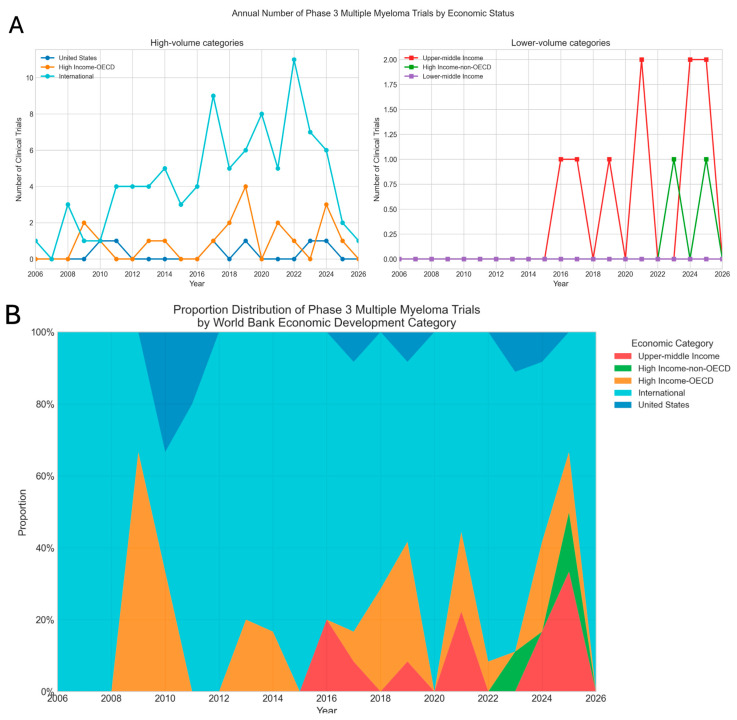
Annual trends in phase 3 multiple myeloma (MM) clinical trials from 2006 to 2026 by economic category. Panel (**A**) shows the annual number of trials, and panel (**B**) shows the proportional contribution of each category per year. Economic categories include the United States (US), high-income OECD countries (HIC-OECD), high-income non-OECD countries (HIC-non-OECD), international trials, upper-middle-income countries (UMIC), and lower-middle-income countries.

**Figure 9:**
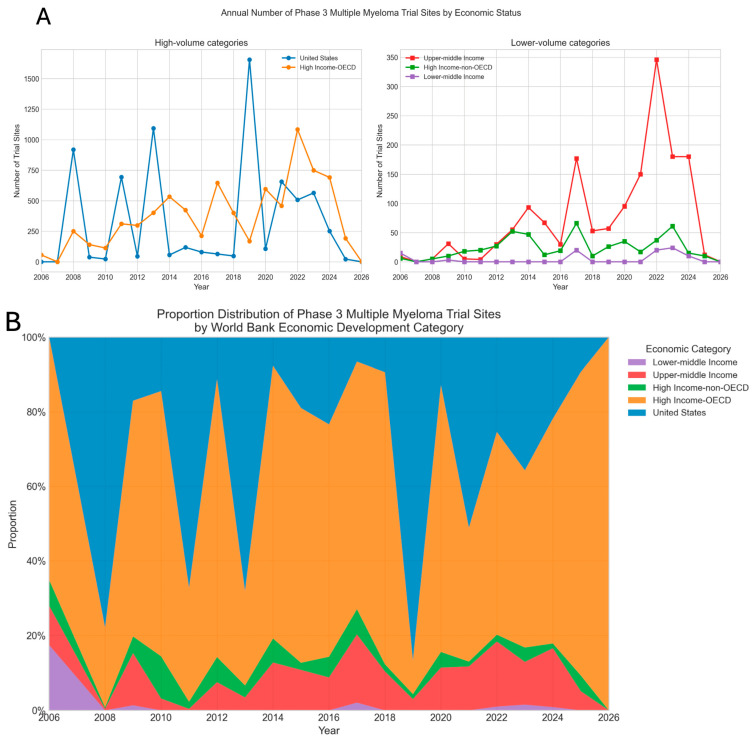
Annual trends in clinical trial sites participating in phase 3 multiple myeloma (MM) trials from 2006 to 2026 by economic category. Panel (**A**) shows the annual number of participating trial sites, and panel (**B**) shows the proportional contribution of each category per year. Economic categories include the United States (US), high-income OECD countries (HIC-OECD), high-income non-OECD countries (HIC-non-OECD), upper-middle-income countries (UMIC), and lower-middle-income countries (LMIC).

Figure 8A,B show the annual number of phase 3 MM clinical trials, and Figure 9 presents the corresponding clinical trial sites over time. As shown in Figure 8, international trials accounted for the largest number of phase 3 trials, followed by HIC-OECD countries, with the US ranking third in terms of trial numbers. Participation from UMICs remained limited, and HIC-non-OECD contributed very few trials throughout the study period.

A different pattern was observed when examining clinical trial sites (Figure 9). Although the number of phase 3 trials was the highest in international studies, the largest number of participating trial sites was contributed by the US and HIC-OECD countries, reflecting their central role in hosting multicenter international phase 3 trials. Both categories showed substantial increases in the number of participating sites during the mid-2010s and the early 2020s. UMICs demonstrated intermittent increases in site participation in later years, whereas HIC-non-OECD and LMICs continued to contribute only a small number of sites.

Overall, although phase 3 trials were largely conducted as international studies, the majority of participating clinical trial sites were in the US and HIC-OECD countries, highlighting their dominant role in hosting large multi-center MM trials. Furthermore. across all phases, early-phase trials were predominantly conducted in the United States and high-income OECD countries, whereas phase 3 trials were more commonly conducted as international multicenter studies.

## 4. Discussion

This analysis of 845 MM clinical trials demonstrates a substantial global increase in trial activity over time, with persistent concentration in the United States and high-income OECD countries. Although international trials have expanded, trial sites and infrastructure remain disproportionately located in these regions. The United States maintained a dominant role in MM clinical research throughout the study period, while HIC-OECD countries contributed an increasing number of trial sites after 2016, indicating broader participation of high-income countries in multicenter trials. Across trial phases, phase 1 trials were mainly conducted in the US and through international collaborations, while phase 2 trials showed a greater contribution from HIC-OECD countries. In phase 3 trials, most studies were conducted as international trials, with the US and HIC-OECD countries hosting most participating sites. Trial density was also highest in high-income countries with smaller populations, whereas large-population countries, such as China, demonstrated lower density despite substantial trial activity. Although UMIC participation increased in recent years, the contributions from HIC-non-OECD countries and LMICs remained limited [13]. In addition, most trials and trial sites were supported by industry sponsors, with relatively few trials funded by public or cooperative research groups.

The marked growth in MM clinical trial activity observed over the study period likely reflects the rapid evolution of the therapeutic landscape. Over the past two decades, successive advances including proteasome inhibitors, immunomodulatory drugs, monoclonal antibodies, bispecific antibodies, and cellular therapies have substantially expanded treatment options and improved patient outcomes. These developments have generated increasing demand for studies evaluating novel agents, combination regimens, treatment sequencing, and earlier disease settings, thereby contributing to the continued expansion and increasing complexity of MM clinical research worldwide.

The rapid increase in trials conducted in upper-middle-income countries represents one of the most notable trends identified in this analysis. Although overall participation remained lower than in high-income regions, the marked growth suggests that several UMICs are becoming increasingly visible in the global MM trial landscape, potentially reflecting expanding oncology infrastructure, increasing research capacity, and greater participation in multinational studies.

Industry sponsors often determine trial locations based on regulatory efficiency, recruitment capacity, and existing research infrastructure; accordingly, sponsor-driven trial design likely contributes to the persistent geographic concentration of MM trials. Despite accounting for over one-third of global trials, the US maintained the highest trial density, whereas large-population countries such as China and India demonstrated substantially lower population-adjusted participation.

Our findings are consistent with prior studies showing that participation in clinical trials and access to new therapies remain uneven across countries. A systematic review of MM trials supporting Food and Drug Administration (FDA) approvals between 2005 and 2019 showed that patient enrollment occurred almost entirely in high-income countries, with no enrollment from low-income countries [7]. The same study also showed that drug approval and access often followed the income level of the countries involved in the trials. Our results support these observations. Across a large registry covering several decades, we found very limited trial activity and very few trial sites in lower-income settings. Other recent studies examining global access to MM therapies have also reported major differences in availability across countries, again suggesting that where trials are conducted strongly influences where new treatments become available [14].

This study provides several additional observations that have been less clearly described in the MM literature. First, by separating trial initiation from trial conduct capacity using measures such as trial sites, site-years, and site density, we show that the concentration of trial sites is even greater than the concentration of trial numbers, especially in the earlier phases of development. Second, the phase-specific analysis shows that although many phase 3 trials are multinational, the trial sites remain concentrated in a limited number of countries. This suggests that multinational trial design does not necessarily mean that research capacity is broadly shared [7]. Although many phase 3 trials were multinational, most trial sites remained concentrated in the United States and other high-income countries, indicating that multinational design often reflects regulatory or enrollment expansion rather than an equitable distribution of research infrastructure. Third, the increasing dominance of industry sponsorship across both trial numbers and trial sites reflects the current structure of MM drug development and shows how sponsor decisions about trial design and site selection influence where research activity takes place and where patients gain access to new therapies [15,16,17].

Several factors likely explain why MM trials and trial sites remain concentrated in high-income settings. Modern MM trials, particularly those evaluating cellular therapies and other immune-based treatments, require specialized centers, close patient monitoring, and strong safety reporting systems [7,14]. These resources are more commonly available in high-income countries, allowing sites to participate repeatedly in clinical trials. In addition, industry sponsors often select trial sites based on operational efficiency, predictable patient recruitment, and established regulatory pathways. This approach favors regions with existing research networks and experienced centers. Previous MM studies also suggest that countries not involved in key clinical trials may experience delayed drug approvals and slower adoption of new therapies, which may further widen differences in access. The temporary decline in trial initiation and site activity around 2020 is consistent with global reports describing widespread disruption of oncology clinical trials during the COVID-19 pandemic [18]. Previous analyses have demonstrated substantial reductions in clinical trial initiation during the first pandemic wave, largely attributable to disruptions in patient recruitment, restrictions on in-person study visits, reallocation of healthcare resources, reduced research capacity, and delays in trial activation and conduct [15,19].

These findings have direct clinical implications. Participation in clinical trials directly influences patient access to experimental therapies and shapes the evidence base guiding treatment guidelines. In regions with limited trial availability, patients may experience delayed access to novel therapies and reduced opportunities to participate in investigational treatment strategies. In addition, evidence generated mainly in high-income countries may not fully represent the clinical and health-system conditions in other settings. The pattern observed in this study, in which international trial numbers increase but trial sites remain concentrated, suggesting that multinational enrollment alone is insufficient to achieve equitable research participation. Addressing these disparities will require targeted measures to expand clinical trial access in underrepresented regions. Potential strategies include investment in diagnostic capacity, development of specialized hematology centers, strengthening of regulatory and ethics-review infrastructure, and creation of regional clinical trial networks that can support safe delivery of novel MM therapies. In addition, multinational trial designs should prioritize broader inclusion of LMIC sites rather than limiting participation to established high-income research centers. Such measures may improve access to emerging therapies, increase the generalizability of trial findings, and reduce global disparities in MM outcomes [20].

Several limitations should be noted. First, this study relies on ClinicalTrials.gov, which may not capture trials registered only in other databases. However, it remains one of the main registries for U.S.-based and multinational trials. Second, information on funding sources and trial sites is based on registry entries and may not always be complete. Third, classifying trials as “international” does not indicate how patients were distributed across countries, and the presence of a trial site does not guarantee equal patient enrollment at each location. Fourth, ClinicalTrials.gov captures trial registration but does not provide detailed data on patient enrollment by country, preventing precise assessment of geographic distribution of enrolled participants. Fifth, registration practices may have varied across regions and over time, particularly in the earlier study period, which could have influenced the observed temporal and geographic distribution of trials.

## 5. Conclusions

In this comprehensive registry-based analysis, global multiple myeloma clinical trial activity expanded substantially between 2006 and 2026 but remained highly concentrated in the United States and other high-income OECD countries across trial numbers, site-years, and population-adjusted density. Although multinational trial designs have increased, the infrastructure required to conduct these studies continues to be anchored in high-income settings, with persistently limited participation from middle- and low-income countries.

## Figures and Tables

**Table 1:** Trials by Economic Status.

Development Status	N Trials (% of Total)	Total Enrollment	Population	Trial Density (/10^6^)	CAGR (%)
United States	337 (39.9%)	17,962.0	341,814,000.0	0.99	9.7
International	271 (32.1%)	73,812.0			9.8
High Income-OECD	129 (15.3%)	12,393.0	789,649,000.0	0.16	8.5
Upper-middle Income	103 (12.2%)	6292.0	2,552,258,000.0	0.04	18.5
High Income-non-OECD	5 (0.6%)	898.0	281,582,000.0	0.02	0.0

N Trials indicates the total number of multiple myeloma clinical trials included in the analysis for each country or economic group. % of Total represents the proportion of all included trials contributed by each category. Total Enrollment reflects the cumulative number of participants enrolled across all trials within each category. Population denotes the total population size corresponding to each country or economic group, based on 2025 United Nations population estimates. Trial Density (/10^6^) represents the number of clinical trials per million population. CAGR (%) indicates the compound annual growth rate of trial counts over the study period. Countries were grouped according to World Bank income classification, with the United States analyzed separately because of its substantially higher trial volume. International trials were defined as trials conducted in two or more countries. Abbreviations: CAGR: Compound annual growth rate.

**Table 2:** Sites by economic status.

Development Status	Number of Countries	Trial Sites (Percentage)	Trial Site Years (Percentage)	Population Thousands	Avg Annual Density	Growth Rate CI
United States	1	9924 (41.31%)	105,262.446 (53.398%)	341,814	14.664	10.3 (−4.0, 26.6)
High Income OECD	31	11,354 (47.26%)	74,165.901 (37.623%)	789,649	4.473	6.4 (−5.1, 19.4)
Upper middle Income	13	2060 (8.57%)	12,989.331 (6.589%)	2,552,258	0.242	10.8 (−1.6, 24.8)
High Income non OECD	8	583 (2.43%)	4022.667 (2.041%)	281,582	0.680	4.2 (−1.6, 10.4)
Lower middle Income	4	104 (0.43%)	687.342 (0.3487%)	2,595,818	0.0126	6.0 (−7.9, 21.9)

Number of Countries indicates the number of distinct countries contributing trial sites within each category. Trial Sites represents the total number of clinical trial sites reported across all trials. Sites (%) indicates the proportion of all trial sites contributed by each category. Trial Site–Years represents the cumulative number of years that trial sites were active, calculated as the product of the number of sites and the duration of trial activity. Site Years (%) indicates the proportion of total trial site–years contributed by each category. Population (thousands) denotes the total population size for each category, expressed in thousands, based on 2025 United Nations population estimates. Average Annual Density represents the mean number of active trial sites per million population per year. Growth Rate CI indicates the compound annual growth rate of site density with corresponding 95% confidence intervals.

**Table 3:** Trial Density of Multiple Myeloma Clinical Trials by Country.

Country	Number of Trials	Total Enrollment	Population 2025	Development Status	Trial Density per Million
United States	337	17,962.0	341,814,000	US	0.99
Israel	6	400.0	9,932,000	HIC-OECD	0.6
Norway	3	215.0	5,550,000	HIC-OECD	0.54
Denmark	3	60.0	5,973,000	HIC-OECD	0.5
Greece	5	219.0	10,246,000	HIC-OECD	0.49
Switzerland	4	411.0	8,968,000	HIC-OECD	0.45
Canada	17	1498.0	41,288,000	HIC-OECD	0.41
Netherlands	6	878.0	17,942,000	HIC-OECD	0.33
France	19	3001.0	68,374,000	HIC-OECD	0.28
Sweden	3	83.0	10,658,000	HIC-OECD	0.28
Ireland	1	16.0	5,281,000	HIC-OECD	0.19
Spain	9	884.0	48,592,000	HIC-OECD	0.19
Japan	20	484.0	123,753,000	HIC-OECD	0.16
Australia	4	363.0	27,184,000	HIC-OECD	0.15
Germany	11	2239.0	84,593,000	HIC-OECD	0.13
United Kingdom	8	1005.0	68,598,000	HIC-OECD	0.12
Austria	1	200.0	9,159,000	HIC-OECD	0.11
Belgium	1	23.0	11,738,000	HIC-OECD	0.09
China	102	6282.0	1,408,112,000	Upper-middle Income	0.07
Italy	4	199.0	58,784,000	HIC-OECD	0.07

Number of Trials indicates the total number of multiple myeloma clinical trials conducted in each country. Total Enrollment reflects the cumulative number of participants enrolled across all trials within each country. Population 2025 denotes the country population size based on 2025 United Nations estimates. Development Status reflects country classification according to World Bank income group, with further stratification by OECD membership where applicable. Trial Density per Million represents the number of clinical trials per million population. Abbreviations: US: United States; HIC-OECD: High-income country—Organisation for Economic Co-operation and Development.

**Table 4:** Sites Density by Country.

Rank	Country	Avg Annual Density	Density	Development Status
1	United States	14.664	0.073	US
2	Israel	12.655	0.491	HIC-OECD
3	Denmark	9.2109	0.0	HIC-OECD
4	Belgium	9.1539	0.0	HIC-OECD
5	Greece	8.850	0.0	HIC-OECD
6	Czech Republic	8.274	0.0	HIC-OECD
7	Spain	8.048	0.0	HIC-OECD
8	Australia	7.356	0.320	HIC-OECD
9	Norway	6.864	0.0	HIC-OECD
10	Puerto Rico	6.770	0.0	HIC-non-OECD
11	Sweden	6.625	0.0	HIC-OECD
12	Austria	6.609	0.0	HIC-OECD
13	France	6.242	0.0	HIC-OECD
14	Netherlands	5.025	0.0	HIC-OECD
15	Canada	4.706	0.0	HIC-OECD
16	Hungary	4.646	0.0	HIC-OECD
17	Italy	4.194	0.0	HIC-OECD
18	Finland	3.933	0.0	HIC-OECD
19	Germany	3.746	0.0	HIC-OECD
20	Ireland	3.721	0.0	HIC-OECD

Rank indicates the country ranking based on average annual trial site density. Average Annual Density represents the mean number of clinical trial sites per million population per year over the study period. Density represents the trial site density per million population in the most recent year of available data. Development Status reflects country classification according to World Bank income group and OECD membership. Abbreviations: US: United States; HIC-OECD: High-income country—Organisation for Economic Co-operation and Development, HIC-non-OECD: High-income country—non-Organisation for Economic Co-operation and Development.

## Data Availability

All data used in this study were obtained from publicly available sources (ClinicalTrials.gov). No new datasets were generated.

## Supplementary Materials

The following supporting information can be downloaded at: https://www.mdpi.com/article/10.3390/curroncol33070396/s1, Table S1: World Bank Income Classification of Countries Based on Gross National Income (GNI) per Capita (2026).

## References

[1] Ebraheem M., Gertz M., Mian H. (2025). Optimizing Multiple Myeloma Clinical Trials: Research Direction, Addressing Limitations, and Strategies for Improvement. Leuk. Lymphoma.

[2] Trends and Charts on Registered Studies|ClinicalTrials.Gov. https://clinicaltrials.gov/about-site/trends-charts.

[3] Sung H., Ferlay J., Siegel R.L., Laversanne M., Soerjomataram I., Jemal A., Bray F. (2021). Global Cancer Statistics 2020: GLOBOCAN Estimates of Incidence and Mortality Worldwide for 36 Cancers in 185 Countries. CA A Cancer J. Clin..

[4] Rajkumar S.V. (2022). Multiple Myeloma: 2022 Update on Diagnosis, Risk-Stratification and Management. Am. J. Hematol..

[5] Sakate R., Fukagawa A., Takagaki Y., Okura H., Matsuyama A. (2018). Trends of Clinical Trials for Drug Development in Rare Diseases. Curr. Clin. Pharmacol..

[6] Zayad A., Ahmed N., Mahmoudjafari Z., Skikne B.S., Lin T., Mushtaq M.U., McGuirk J., Lutfi F., Bhurtel E., Bhutani M. (2025). The Impact of Sponsors on Development of Clinical Research in Multiple Myeloma and AL Amyloidosis: An In-Depth Analysis. Clin. Lymphoma Myeloma Leuk..

[7] Fatoki R.A., Koehn K., Kelkar A., Al Hadidi S., Mehra N., Mian H., Landgren O., Kazandjian D., Hoffman J., Sborov D.W. (2022). Global Myeloma Trial Participation and Drug Access in the Era of Novel Therapies. JCO Glob. Oncol..

[8] Drain P.K., Parker R.A., Robine M., Holmes K.K. (2018). Global Migration of Clinical Research during the Era of Trial Registration. PLoS ONE.

[9] World Bank Country and Lending Groups—World Bank Data Help Desk. https://datahelpdesk.worldbank.org/knowledgebase/articles/906519-world-bank-country-and-lending-groups.

[10] Members and Partners. https://www.oecd.org/en/about/members-partners.html.

[11] World Urbanization Prospects. https://population.un.org/wup/.

[12] World Urbanization Prospects 2025. https://population.un.org/wup/assets/Publications/undesa_pd_2025_wup2025_summary_of_results_final.pdf.

[13] Snyder R.A., Hillman S.L., Marcotte V., Paskett E.D., George S., Hahn O., Mandrekar S.J. (2024). Impact of the COVID-19 Pandemic on Cancer Clinical Trials: Alliance for Clinical Trials in Oncology Experience (Alliance A152022). J. Clin. Trials.

[14] Atallah R., Shatnawi Y., Ayoobkhan M.F.S., Saif M.S.I., Naeem M., Logan E., Mushtaq M.U., Rana S., Mammadzadeh A., Hashmi S.K. (2025). Global Access to Multiple Myeloma Therapies. JCO Glob. Oncol..

[15] Upadhaya S., Yu J.X., Oliva C., Hooton M., Hodge J., Hubbard-Lucey V.M. (2020). Impact of COVID-19 on Oncology Clinical Trials. Nat. Rev. Drug Discov..

[16] Fernandes L.L., Zhou J., Kanapuru B., Horodniceanu E., Gwise T., Kluetz P.G., Bhatnagar V. (2021). Review of Patient-Reported Outcomes in Multiple Myeloma Registrational Trials: Highlighting Areas for Improvement. Blood Cancer J..

[17] Salek S., Ionova T., Oliva E.N., Andreas M., Skoetz N., Kreuzberger N., Laane E. (2022). The Reporting, Use, and Validity of Patient-Reported Outcomes in Multiple Myeloma in Clinical Trials: A Systematic Literature Review. Cancers.

[18] Unger J.M., Xiao H. (2021). The COVID-19 Pandemic and New Clinical Trial Activations. Trials.

[19] Nishiwaki S., Ando Y. (2021). COVID-19 Pandemic and Trends in Clinical Trials: A Multi-Region and Global Perspective. Front. Med..

[20] Fundytus A., Sullivan R., Vanderpuye V., Seruga B., Lopes G., Hammad N., Sengar M., Hopman W.M., Brundage M.D., Booth C.M. (2017). Delivery of Global Cancer Care: An International Study of Medical Oncology Workload. J. Glob. Oncol..

