# Supplementary material for: The ClinicalTrials.gov Landscape of Multiple Myeloma Clinical Trials: A 20-Year Analysis of Geographic Distribution and Growth Patterns: USMIRC Analysis
Source: Curr Oncol. 2026 Jul 1;33(7):396. doi: 10.3390/curroncol33070396 (PMC13408958; doi:10.3390/curroncol33070396)
Supplement: Supplementary file 1 [file curroncol-33-00396-s001.zip › curroncol-4328716-supplementary.pdf]

Table S1: World Bank Income Classification of Countries Based on Gross National Income (GNI) per Capita (2026).

	GNI per capita (USD, 2026)
Low-Income economies	1,135 or less
Lower-middle-income economies	1,136 - 4,495
Upper-middle-income economies	4,496 - 13,935
High-income economies	greater than 13,935

Table S1. World Bank income classification of countries according to gross national income (GNI) per capita for the 2026 fiscal year. Countries were categorized as low-income, lower-middle-income, upper-middle-income, or high-income based on the World Bank Atlas method thresholds. These classifications were used to assign the development status of countries included in this study.
